# Live human metapneumovirus vaccine candidates attenuated by temperature sensitivity mutations from human respiratory syncytial virus

**DOI:** 10.1128/jvi.00692-26

**Published:** 2026-07-06

**Authors:** Pramila Lamichhane, Lijuan Yang, Celia Santos, Hong-Su Park, Li Ou, Tongqing Zhou, Cyril Le Nouën, Ursula J. Buchholz

**Affiliations:** 1 RNA Viruses Section, Laboratory of Infectious Diseases, National Institute of Allergy and Infectious Diseases, National Institutes of Health 35037, Bethesda, Maryland, USA; 2 Vaccine Research Center, National Institutes of Health 2511, Bethesda, Maryland, USA; Loyola University Chicago - Health Sciences Campus, Maywood, Illinois, USA

**Keywords:** human metapneumovirus, temperature sensitivity, live-attenuated vaccine, intranasal immunization

## Abstract

Human metapneumovirus (HMPV, genus *Metapneumovirus*) and respiratory syncytial virus (HRSV, genus *Orthopneumovirus*) are pediatric respiratory pathogens of the *Pneumoviridae* family. We previously developed live-intranasal HRSV vaccine candidates attenuated by genetically stabilized temperature sensitivity/attenuating (*ts/att*) mutations in the HRSV polymerase (L) ORF, including sites “*1030s*” (L codon changes S1313_(AGC-to-TCA)_/Y1321K_(AAA)_, named after clone number 1030), and “Δ1313/I1314L,” comprising a genetically stabilized deletion of L codon 1313. In pediatric vaccine studies, HRSV candidates with “*1030s*” or Δ1313/I1314L were attenuated, immunogenic, and genetically stable. Given the similarities between HRSV and HMPV, we introduced corresponding modifications into the HMPV L ORF, resulting in HMPV with deletion of codon 1238 (Δ1238), corresponding to HRSV Δ1313/I1314L, and HMPV with C1238S_(TCA)_/Y1246K_(AAA)_ substitutions together (C1238S/Y1246K, corresponding to HRSV “*1030s*”), or individually (C1238S, Y1246K), serving as experimental controls. All versions replicated well in Vero cells at 32°C. At 37°C, replication of Δ1238, C1238S/Y1246K, and Y1246K was delayed, and peak titers were ~80-fold, 200-fold, and 25-fold lower than wild-type HMPV. As predicted, the C1238S control was not temperature-sensitive (shutoff temperature [*T*_SH_] > 40°C, similar to wild-type HMPV), while Y1246K and Δ1238 (*T*_SH_ = 39°C) and C1238S/Y1246K (*T*_SH_ = 38°C) had a *ts* phenotype. Thus, the addition of the C1238S_(TCA)_ substitution increased the temperature sensitivity of Y1246K. The HMPV *ts/att* sites were genetically stable under temperature stress. In hamsters inoculated intranasally, C1238S/Y1246K and Δ1238 were restricted in replication, yet immunogenic and protective against HMPV challenge. Thus, inclusion in the HMPV L ORF of C1238S/Y1246K and Δ1238 mutations, corresponding to HRSV *ts/att* sites, provided genetically stable live-attenuated HMPV, suitable for clinical development.

**IMPORTANCE:**

Human metapneumovirus (HMPV) is a leading cause of lower respiratory illness in young children, and there are no licensed vaccines. Similarly to immunization strategies against the related human respiratory syncytial virus (HRSV), live-attenuated HMPV vaccines for intranasal immunization would be most suitable for young children. Live-intranasal vaccines infect and induce immunity in the presence of residual maternal antibodies without priming for enhanced respiratory disease. Genetic stability of attenuating mutations represents a challenge in vaccine design. We generated live-attenuated HMPV vaccine candidates by introducing into corresponding sites of the HMPV polymerase gene temperature-sensitivity-inducing attenuating mutations that had been originally developed for HRSV, genetically stabilized, and proven to be free of reversions in HRSV vaccine studies. The resulting HMPV vaccine candidates are temperature-sensitive, attenuated, and genetically stable under temperature stress. In the hamster model, the new HMPV candidates were immunogenic and protective. These live-attenuated HMPV candidates will be advanced to pediatric vaccine studies.

## INTRODUCTION

**Fig 1:**
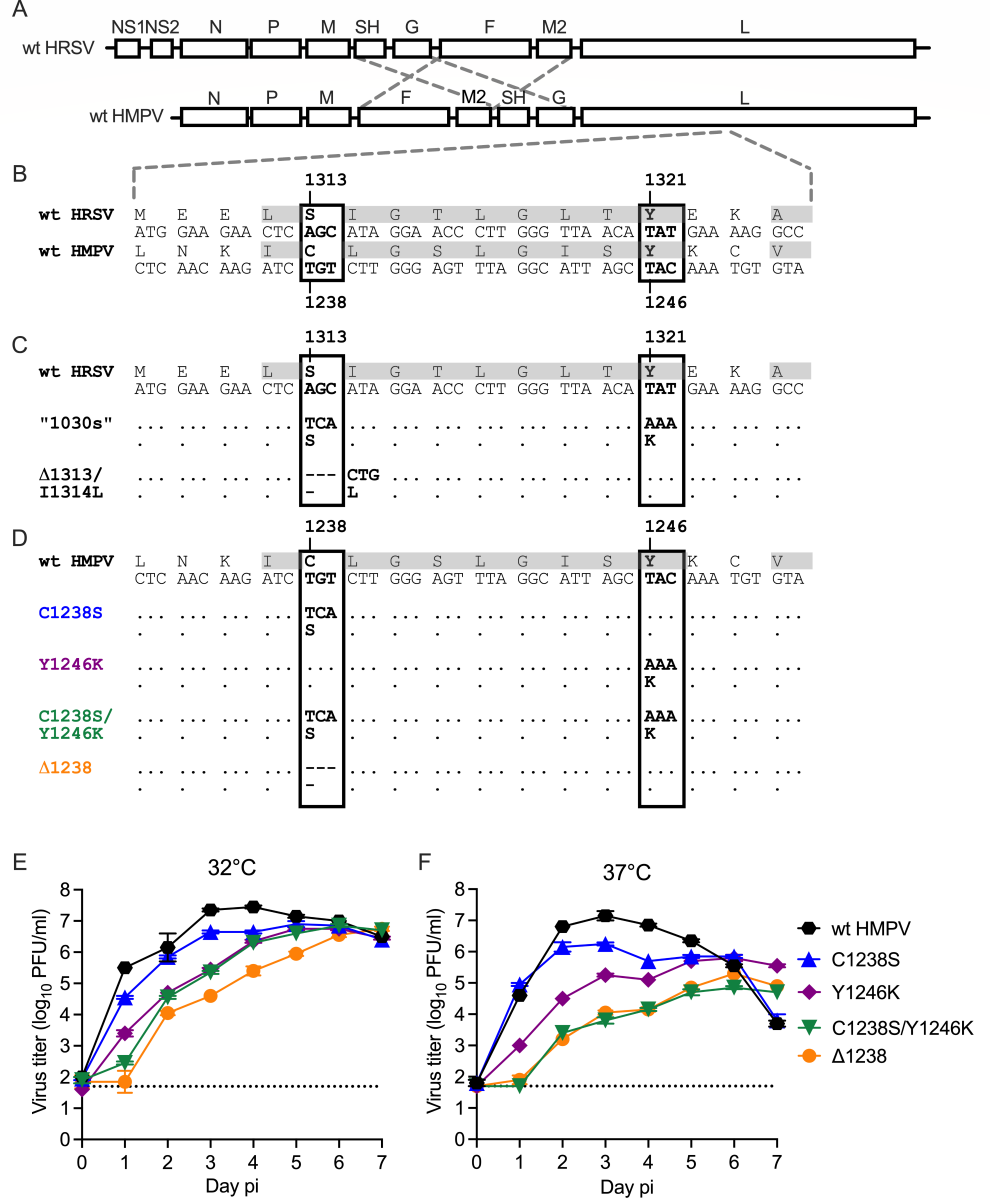
Genome organization of HMPV mutants and replication in Vero cells. (**A**) Genome maps and (**B**) enlargement of aligned regions of the L ORFs of HMPV and HRSV. The genome maps show genes encoding nonstructural proteins NS1, NS2 (HRSV only), nucleoprotein N, phosphoprotein P, matrix protein M, and polymerase protein L (HMPV and HRSV). The order of the small hydrophobic protein (SH) and attachment glycoprotein G genes and the glycoprotein F and M2 genes is inverted in HRSV and HMPV (indicated by diagonal lines). The L genes of HRSV and HMPV share amino acid (aa) similarities of 45%. A region spanning nt 10832–10879 of HMPV (L codons 1222–1265 of strain CAN97-83, GenBank accession number AY297749) and nt 12423 through 12470 of HRSV (L codons 1297–1340 of strain A2, GenBank accession number KT992094) is shown in panel B, with amino acid similarities highlighted in gray. HRSV L codons 1313 and 1321, the sites of previously identified *ts/att* mutations from well-characterized HRSV vaccine candidates, and the corresponding HMPV L codons 1238 and 1246 are framed. (**C**) The same HRSV L coding region as in panel B, with the nucleotide and amino acid substitutions of HRSV *ts/att* mutations “*1030s*” and Δ1313/I1314L indicated. (**D**) Enlargement of the L gene region of the four newly created HMPV mutants with the indicated mutations. (**E and F**). Multicycle replication kinetics in Vero cells. Sub-confluent wells of Vero cells in six-well plates were infected with the indicated viruses at an MOI of 0.01 PFU/cell and incubated at 32°C (**E**) and 37°C (**F**). At 24-h intervals, duplicate wells of Vero cells infected with the indicated viruses were scraped, vortexed briefly, and supernatants were clarified by low-speed centrifugation. Aliquots were snap frozen on dry ice. Virus titers were determined later by immunoplaque assay at 32°C. Titers correspond to the mean of two replicate titrations from two replicate wells at each time point. Day 0 titers correspond to the back titration of the inocula.

Human metapneumovirus (HMPV) is a leading cause of acute respiratory illness in infants, children, the elderly, and immunocompromised individuals. HMPV was first described in 2001 (1) and accounts for about 11% of acute lower respiratory illness (ALRI) among children under 5 years of age globally (2). Based on global estimates for the year 2018, HMPV is associated with 14.2 million ALRI cases annually in children younger than 5 years, including about 500,000 hospital admissions and 11,300 deaths (3). In this age group, the overall burden of HMPV respiratory illness is exceeded only by that of human respiratory syncytial virus (HRSV). Primary infections with HMPV occur slightly later in life than primary HRSV infections, and more than 90% of children have experienced an HMPV infection by age 5 (4, 5). Clinical manifestations of HMPV illness resemble those caused by HRSV, ranging from mild respiratory illness to bronchiolitis and pneumonia (2). HMPV, genus *Metapneumovirus*, and HRSV, genus *Orthopneumovirus*, both belong to the family *Pneumoviridae* and share similarities in genome organization and structure (6) (Fig. 1A). Both contain a single-stranded negative-sense RNA genome. The HMPV genome of about 13.3 kb comprises eight genes in the order of 3′-N-P-M-F-M2-SH-G-L-5′, encoding nine proteins. The HRSV genome of 15.2 kb includes two additional genes that encode non-structural proteins NS1 and NS2 (gene order 3′-NS1-NS2-N-P-M-SH-G-F-M2-L-5′, with the order of the SH and G genes and F and M2 genes reversed compared to HMPV) (1, 7) (Fig. 1A). HMPV and HRSV both encode three surface glycoproteins, the small hydrophobic protein (SH), fusion (F), and attachment (G) proteins. For HRSV, both the G and F proteins represent major protective antigens, while for HMPV, the F protein was recognized as the major protective antigen; its G protein is not a major protective antigen, at least in animal models (8). As is typical for negative-strand RNA viruses, the HMPV RNA is tightly encapsidated by the nucleoprotein (N), which, together with the phosphoprotein (P) and polymerase (L) protein, forms the ribonucleoprotein complex, the minimal transcription/replication unit. The amino acid identities of the HMPV and HRSV L ORFs are substantial (45%), and based on sequence-relatedness and cryo-electron microscopy reconstructions, the functional domains of the HMPV and HRSV L proteins are structurally similar (9, 10).

Despite the substantial burden of HMPV illness, particularly in the pediatric population, no licensed vaccines, prophylactic antibodies, or antivirals have been approved for HMPV to date. In the case of HRSV, two extended half-life monoclonal antibodies were licensed in 2023 (nirsevimab) (11) and 2024 (clesrovimab) (12) for passive prophylaxis against HRSV illness in infants under 8 months of age born during or entering their first HRSV season; nirsevimab is also available for infants and children aged 8-19 months who are at increased risk of severe RSV disease entering their second RSV season. A maternal HRSV F subunit vaccine to protect infants during their first HRSV season was licensed in 2023 (13). Monoclonal antibodies for passive prophylaxis against HMPV are in development as well, targeting the HMPV F protein, the major protective HMPV antigen (14–16), but no HMPV antibody has received FDA approval yet. Compared to HRSV, HMPV infects slightly later in life; since the window of protection of maternal antibodies against HMPV does not fully overlap with the age group at highest risk for severe HMPV illness (17), a maternal HMPV vaccine would likely be less effective against severe respiratory illness than the maternal HRSV vaccine. In any case, similarly to HRSV, passive immunity will wane; safe and effective vaccines for active immunization against both viruses continue to be under development to address the substantial burden of respiratory illness in older infants and children under 5 years of age.

In animal models, parenteral immunization of HMPV naïve recipients with formalin-inactivated HMPV primed for markers of disease enhancement, similar to HRSV (18, 19), while intranasal immunization with attenuated HMPV was not associated with markers for enhancement (19). A pediatric vaccine development program based on HMPV F mRNA was recently stopped following a safety signal in HMPV-naïve vaccine recipients; compared to placebo recipients, a higher number of vaccine recipients experienced severe HMPV illness during the subsequent HMPV season (the program also included an HRSV mRNA vaccine, with similar results in HRSV-naïve recipients). Underlying mechanisms are currently being studied (20). Thus, subunit or non-replicating HMPV vaccines remain contraindicated for young children. In the case of HRSV, live-attenuated or live-vectored vaccines for intranasal immunization are not associated with a risk of priming for vaccine-enhanced disease in virus-naïve vaccinees that are subsequently exposed to community-acquired infection (21–23), and this can be extended to HMPV based on the close similarity of these viruses and their pathogenesis. Past efforts to generate live intranasal HMPV vaccine candidates have relied on attenuation by deletion of accessory proteins such as G, SH, and M2-2, either individually or in combination (24–26), or on a “modified Jennerian” approach, involving the replacement of the HMPV N and/or P genes with those of an avian MPV counterpart (27). The development of live-vectored vaccines has focused on the HMPV F protein, the major protective HMPV antigen (28); vectored vaccine candidates based on bovine/human parainfluenza virus type 3 expressing the HMPV F protein are in development as bivalent HPIV3/HMPV vaccines (29, 30), and a bivalent HRSV/HMPV vaccine candidate was described, based on HMPV expressing the HRSV F antigen from an additional gene (31).

While live-vectored vaccines expressing the HMPV F protein could be useful as stand-alone intranasal vaccines or in a heterologous prime/boost approach in combination with live-attenuated HMPV, for primary immunization in early childhood, a vaccine candidate based on live-attenuated HMPV would be most desirable. Live-attenuated HMPV vaccines mimic natural infection and elicit robust active immunity without requiring adjuvant. Unlike subunit or vectored vaccines that rely on the HMPV F protein alone, live-attenuated HMPV incorporates the full complement of HMPV T- and B-cell epitopes (32, 33), expected to elicit protective humoral and cellular immune responses. Vaccine candidates with temperature-sensitivity phenotypes provide added safety against reactogenicity at the core body temperature of the lower respiratory tract. To generate live-attenuated vaccine candidates against other respiratory pathogens, it was possible to transfer attenuating mutations to corresponding regions of related viruses, guided by sequence alignments (34–36). For HMPV, this strategy has also been explored in prior studies (37, 38).

In the case of HRSV, the development of cDNA-derived live-attenuated vaccine candidates with temperature sensitivity (*ts*) mutations has a long history. A highly attenuated and temperature-sensitive predecessor of current HRSV lead candidates, rA2/cp/ΔSH/*248*/*1030*, was safe and immunogenic in young RSV-seronegative children and 1- to 2-month-old infants (39), but *in vitro* analysis of nasal wash specimens from vaccine recipients revealed that about one-third of vaccine isolates exhibited a partial loss of the *ts* phenotype. Sequence analysis confirmed that some isolates had lost one of two *ts* mutations that were incorporated in the L ORF of rA2/cp/ΔSH/*248*/*1030*, namely either the Q831L (“*248*”) or Y1321N (“*1030*”) mutations, with “*248*” and “*1030*” referring to plaque identities rather than amino acid positions (40). Subsequently, both sites were extensively studied preclinically and stabilized by reverse genetics against any codon change that would result in loss of attenuation or temperature sensitivity (41, 42). The stabilization of the HRSV “*1030*” *ts/att* site Y1321N at amino acid (aa) 1321 of the HRSV L ORF was based on a tyrosine-to-lysine substitution (Y1321K_(AAA)_; Fig. 1B and C), in conjunction with a synonymous substitution of a serine codon S1313_(AGC-to-TCA)_ located eight codons upstream (42) (Fig. 1C, middle line). The 1321K_(AAA)_ attenuating mutation is refractory to de-attenuation, since there is no single-nucleotide (nt) change that would result in reversion to the original Y1321 amino acid assignment, or any other among the 20 possible amino acid assignments supporting a de-attenuating phenotype (42). The S1313_(AGC-to-TCA)_ codon substitution was designed to prevent a second-site compensatory S_(AGC)_1313C_(TGC)_ point mutation (underlined) that readily occurred during replication of “*1030*” viruses under temperature pressure, reversing the attenuation and temperature sensitivity phenotype conferred by the Y1321K substitution (42). Of note, while the S_(AGC)_1313C_(TGC)_ mutation readily occurred and was de-attenuating in HRSV with the Y1321K_(AAA)_ substitution, an S_(AGC)_1313C_(TGC)_ substitution had no detectable phenotypic effect when introduced on its own into recombinant wt HRSV (bearing a Y1321 assignment). The genetically stabilized *ts/att* site S1313_(TCA)_/Y1321K_(AAA)_, termed “*1030s*,” confers stable attenuation and temperature sensitivity to HRSV vaccine candidates (shutoff temperature for HRSV plaque formation [*T*_SH_] of 38°C compared to >40°C for wt HRSV) (42).

In the context of studies to stabilize the “*1030*” site, deletion of codon S1313 (Δ1313) of the HRSV L protein was identified as another promising *ts/att* mutation (Fig. 1C, bottom), conferring attenuation and a *T*_SH_ of 37°C. Under temperature stress, HRSV Δ1313 readily acquired a non-synonymous I_(ATA)_1314T_(ACA)_ point mutation (nt change underlined) in the L codon adjacent to the codon deletion, which compensated for the attenuation and temperature sensitivity mediated by the S1313 codon deletion (43). To prevent any single-nucleotide mutation resulting in an I1314T substitution, an I_(ATA)_1314L_(CTG)_ substitution (nt changes underlined) was added, successfully stabilizing HRSV vaccine candidates attenuated by deletion of codon S1313. Thus, two promising genetically stabilized *ts/att* sites in the L protein have emerged from these HRSV vaccine studies, “*1030s*” [S1313_(TCA)_/Y1321K_(AAA)_] and Δ1313/I1314L_(CTG)_ (Fig. 1C). HRSV vaccine candidates containing “*1030s*” (22, 44–46) or Δ1313/I1314L (22, 47, 48) have since been evaluated in several clinical studies. No genetic instability at these attenuating sites has been detected to date. Given the structural and sequence similarities between the L proteins of HRSV and HMPV, we generated HMPV vaccine candidates with changes corresponding to the HRSV “*1030s*” and Δ1313/I1314L sites and characterized their phenotypes *in vitro* and in the hamster model.

## RESULTS

### HMPV with amino acid substitutions in the L ORF corresponding to temperature sensitivity/attenuating (*ts/att*) sites from HRSV vaccine candidates

To generate HMPV vaccine candidates with *ts/att* mutations, we selected two genetically stabilized sites in the polymerase (L) protein from lead HRSV *ts/att* vaccine candidates (Fig. 1C), prioritizing *ts/att* sites that are well characterized with respect to temperature sensitivity, attenuating effects, and genetic stability in clinical studies (44–48). The first of these genetically stabilized HRSV *ts/att* sites, designated “*1030s*” (42), represents a Y1321K_(AAA)_ substitution in the HRSV L ORF in conjunction with a synonymous codon replacement at S1313 (AGC to TCA, Fig. 1C, middle). The synonymous replacement S1313_(AGC-to-TCA)_ prevented the frequently occurring compensatory S_(AGC)_1313C_(TGC)_ point mutation (nt change underlined) that caused de-attenuation of HRSV attenuated by Y1321K_(AAA)_ substitution (42). In HRSV vaccine candidates, the stabilized site “*1030s*” [S1313_(TCA)_/Y1321K_(AAA)_] confers stable attenuation and temperature sensitivity (*T*_SH_ = 38°C [42]). The second HRSV *ts/att* site, Δ1313/I1314L, refers to a deletion of the HRSV L codon 1313, in conjunction with the I_(ATA)_1314L_(CTG)_ stabilizing substitution (nt changes underlined; Fig. 1C, bottom) and confers stable attenuation and a T_SH_ for HRSV plaque formation at 37°C (43).

Alignment of the amino acid sequences of the L proteins of HRSV and HMPV revealed substantial similarities in the region of these HRSV *ts/att* sites (Fig. 1A and B). Codon S1313_(AGC)_ in the wt HRSV L ORF corresponds to codon C1238_(TGT)_ in the wt HMPV L ORF; the Y1321_(TAT)_ amino acid assignment in wt HRSV L ORF is conserved in HMPV, corresponding to codon Y1246_(TAC)_ in the wt HMPV L ORF. To study substitutions corresponding to the *ts/att* HRSV sites, four versions of recombinant HMPV were designed: (i) HMPV C1238S_(TCA)_ (C1238S, Fig. 1D, second line) with a non-synonymous substitution in the L ORF corresponding to the second-site stabilizing assignment S1313_(TCA)_ from HRSV “*1030s.*” In previous work, when introduced on its own into recombinant HRSV to replace the wt HRSV S1313_(AGC)_ assignment, neither the synonymous S1313_(TCA)_ nor the non-synonymous S1313C_(TGC)_ change had any detectable phenotypic effect (42), and thus we predicted for HMPV no or minimal phenotypical consequences of the non-synonymous C_(TGT)_1238S_(TCA)_ substitution (nt changes underlined). (ii) Y1246K (Fig. 1D, third line) included a Y_(TAC)_1246K_(AAA)_ substitution, corresponding to the HRSV *ts/att* substitution Y_(TAT)_1321K_(AAA)_ that in HRSV under temperature stress exhibited a partial phenotypic reversion by the compensatory S1313C mutation. Since HMPV naturally encodes a cysteine residue at position 1238 (8 amino acids N-terminally from Y1246K) that, in analogy to HRSV, might compensate for the predicted *ts/att* effect of the Y1246K substitution, the *ts/att* effect of Y1246K alone on HMPV was expected to be smaller than that observed for Y1321K on HRSV. (iii) For a stronger *ts/att* effect on HMPV, a C_(TGT)_1238S_(TCA)_ substitution was combined with Y_(TAC)_1246K_(AAA)_ (C1238S/Y1246K; Fig. 1D, fourth line), providing for a genetically stabilized cysteine-to-serine substitution eight amino acids upstream of the main attenuating change Y1246K and corresponding to HRSV with “*1030s*” mutation. (iv) Finally, we designed a version of HMPV with a deletion of codon 1238 (Δ1238; Fig. 1D, bottom), corresponding to the *ts/att* deletion of codon 1313 of the HRSV L protein. Since HMPV naturally contains an L_(CTT)_ codon at position 1239, that is, the next codon following C1238, no single-nucleotide change would result in a potentially compensatory threonine mutation that had emerged under temperature stress in HRSV at this adjacent position. Thus, further stabilization of HMPV L codon L1239_(CTT)_ was not deemed necessary (Fig. 1D). cDNAs encoding the HMPV-SHs full-length antigenome sequences (based on strain CAN97-83, with a codon-stabilized version of the small hydrophobic [SHs] gene [49]) were modified by site-directed mutagenesis to introduce the nt substitutions in the L gene shown in Fig. 1D.

### Recovery from cDNA and characterization of replication and temperature sensitivity of HMPV mutant viruses in cell culture

Four versions of recombinant HMPVs with the modifications shown in Fig. 1D were readily recovered by reverse genetics (see Materials and Methods). Working P2 stocks were generated on Vero cells. The consensus sequence of P2 stocks was verified by direct sequencing of overlapping PCR products covering the entire genomes (see Materials and Methods), confirming that each virus was free of adventitious mutations.

Next, we evaluated the multicycle replication of these viruses in Vero cells at the fully permissive temperature of 32°C, simulating the lower temperature of the upper respiratory tract, as well as at 37°C, simulating physiological body temperature (Fig. 1E and F). Vero monolayers were inoculated with the indicated viruses at the low multiplicity of infection (MOI) of 0.01, and replication was monitored for 7 days. All four recombinant viruses replicated to high titers at 32°C (Fig. 1E). Replication of wt HMPV was most efficient, with peak titers of 7.5 log_10_ PFU/mL by day 4 post-infection (pi), whereas replication of HMPV bearing the C1238S or the *ts/att* assignments from HRSV reached peak titers that were only four- to fivefold lower, and their replication was slightly delayed compared to wt HMPV (6.9 log_10_ PFU/mL for C1238S on day 5; 6.8 and 6.9 log_10_ PFU/mL, respectively, for Y1246K and C1238S/Y1246K on day 6; and 6.8 log_10_ PFU/mL for Δ1238 on day 7). Importantly, the *ts/att* viruses replicated to levels suitable for vaccine manufacture on Vero cells at permissive temperatures.

At 37°C, wt HMPV reached peak titers of 7.2 log_10_ PFU/mL on day 3. Titers of C1238S also peaked on day 3 but remained about 10-fold lower than wt HMPV. On the other hand, replication of Y1246K, C1238S/Y1246K, and Δ1238 was delayed, peaking on day 6; peak titers were about 25-fold (Y1246K), 200-fold (C1238S/Y1246K), and 80-fold (Δ1238) lower compared to wt HMPV. On day 3, when wt HMPV replication at 37°C peaked, titers of Δ1238 and C1238S/Y1246K were still >1,000-fold lower (Fig. 1F). Thus, the *in vitro* replication patterns of Δ1238 and C1238S/Y1246K were comparable, with lower efficiency of replication at 37°C than at 32°C, suggesting that these viruses might be temperature sensitive.

To determine the level of temperature sensitivity, the viruses were evaluated on Vero cells for their ability to form plaques at temperatures ranging from 32°C to 40°C (Table 1). The shutoff temperature *T*_SH_ is defined as the lowest restrictive temperature at which there is a reduction in plaque number compared to 32°C, that is, 100-fold or greater than that observed for wt HMPV at the two temperatures. The *ts* phenotype is defined as having a *T*_SH_ of 40°C or less. As expected, temperatures of 32–40°C did not have a significant effect on plaque formation of wt HMPV or a GFP-expressing version of HMPV (rHMPV-GFP) that was included as an additional control (Table 1). As predicted based on wt HRSV that naturally has a serine assignment at the corresponding L codon 1313 and tolerates an S1313C substitution without phenotypic effect (42), C1238S readily formed plaques over the temperature range, indicating a *T*_SH_ > 40°C and confirming a similar tolerance for serine or cysteine at the corresponding L position 1238. HMPV with Y1246K alone, with the wt HMPV cysteine assignment at L codon 1238, had a *T*_SH_ of 39°C (Table 1), indicating a moderate *ts* phenotype, 1°C lower than that observed for HRSV with Y1321K and compensatory S1313C mutation (*T*_SH_ = 40°C [42]). Inclusion of C1238S together with Y1246K in HMPV (C1238S/Y1246K) further reduced the *T*_SH_ to 38°C, revealing a level of temperature sensitivity similar to that of HRSV “*1030s*” with stabilized S1313_(TCA)_ and Y1321K_(AAA)_ assignments in its L protein (42). In addition, HMPV C1238S/Y1246K had a micro-plaque phenotype at ≥37°C (Table 1). Thus, similarly to HRSV that accommodates serine or cysteine residues individually at L position 1313 with no effect on temperature sensitivity, the C1238S substitution alone does not confer temperature sensitivity to HMPV. Similar to HRSV “*1030s*,” in HMPV, the presence of C1238S in addition to Y1246K further increases temperature sensitivity conferred by Y1246K.

**TABLE 1:** Temperature sensitivity and attenuation of HMPV vaccine candidates bearing *ts/att* mutations identified in HRSV vaccine candidates

Virus	Virus titer (PFU per mL) at indicated temperature (°C)^*a*^								
	32	35	36	37	38	39	40	*T*_SH_ ^*b*^	*T*_M_^*c*^
wt HMPV	7.7	7.6	7.7	7.6	7.6	7.6	7.4	>40	>40
rHMPV-GFP	6.6	6.2	6.3	6.3	6.2	5.9	5.4	>40	>40
C1238S	6.5	6.5	6.5	6.4	5.8	5.8	5.8	>40	>40
Y1246K	6.4	6.4	5.8	5.6	5.4	***3.9*** *^b^* ^,*c*^	*2.4*	**39**	**39**
C1238S/Y1246K	6.3	5.7	5.7	*5.3*	***3.5***	*2.4*	*2.3*	**38**	**37**
Δ1238	6.5	5.7	5.5	4.8	4.6	***2.7***	*2.3*	**39**	**39**

^*a*^ The temperature sensitivity (*ts*) phenotype for each virus was evaluated by plaque assay on Vero cells at the indicated temperatures. For viruses with a *ts* phenotype, the shutoff temperatures (*T*_SH_) and micro-plaque temperatures (*T*_M_) are indicated in boldface.

^*b*^ Shutoff temperature (*T*_SH_, underlined) is defined as the lowest restrictive temperature at which the reduction compared to 32°C is 100-fold or greater than that observed for wt RSV at the two temperatures. The *ts* phenotype is defined as having a shutoff temperature of 40°C or less.

^*c*^ *T*_M_, micro-plaque temperature (italics) is defined as the lowest restrictive temperature at which the micro-plaque phenotype (compared to the same virus at 32°C) is observed.

HMPV with deletion of codon 1238 (Δ1238) was also temperature sensitive, with a *T*_SH_ of 39°C (Table 1), but slightly less restricted than C1238S/Y1246K (*T*_SH_ = 38°C).

### Phenotypic and genetic stability of HMPV L mutants at permissive and increasingly restrictive temperatures (*in vitro* stress test)

**Fig 2:**
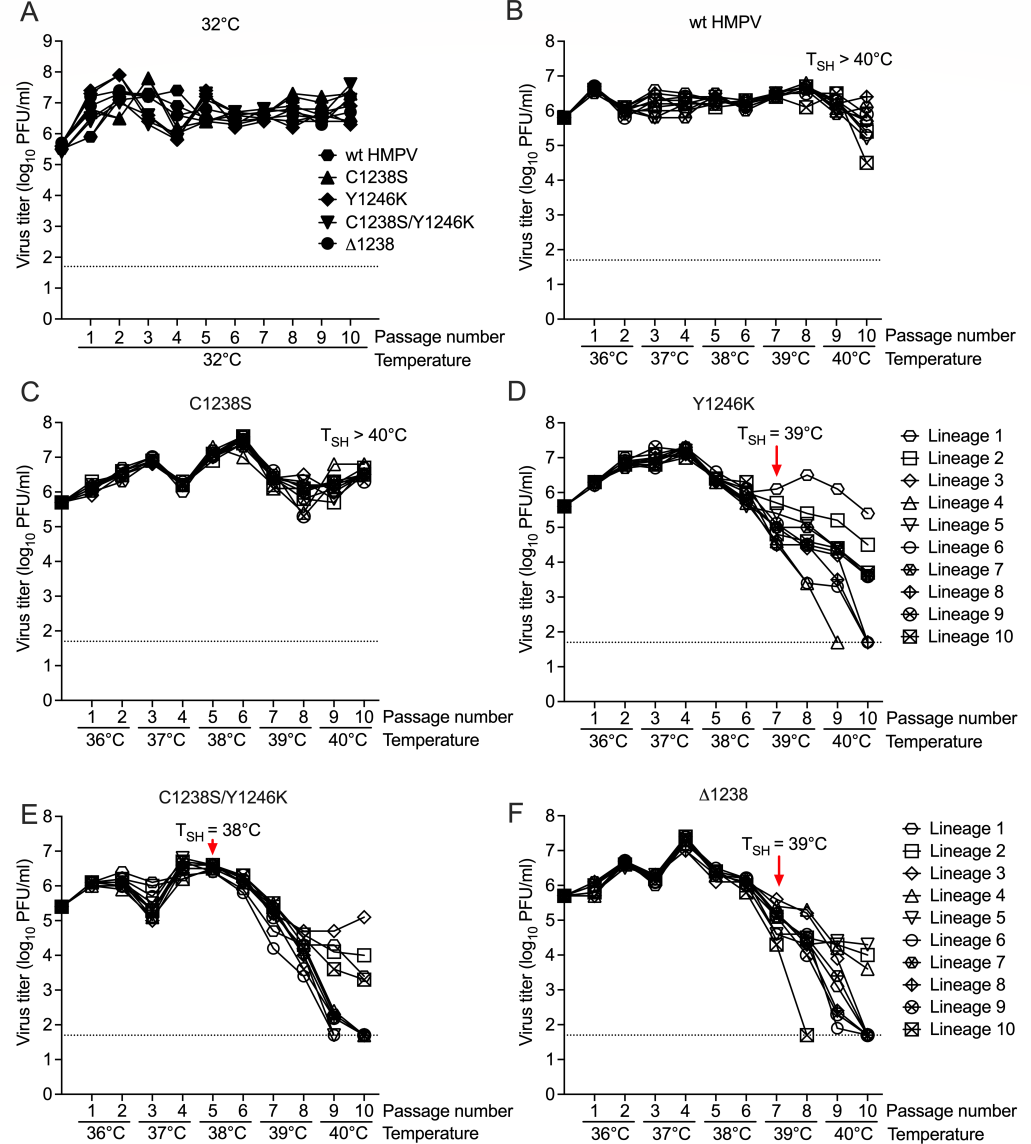
*In vitro* stress test of HMPV mutants. Vero cells in 25 cm^2^ flasks were infected with the indicated viruses at an MOI of 0.1 PFU/cell. Twelve flasks for each virus were used, each flask representing a separate lineage. (**A**) Two flasks (lineages) were serially passaged at the permissive temperature of 32°C for 10 passages, serving as control. (**B–F**) Ten flasks were subjected to serial passage at increasing temperatures, with an increase in the incubation temperature by 1°C every second passage from 36°C to 40°C, for a total of 10 passages. Infected cells were monitored for cytopathic effect and typically harvested between 4 and 7 days post-infection. Infected cells were scraped into the medium, vortexed briefly, and supernatants were clarified by centrifugation at low speed. Aliquots were snap frozen in dry ice and used for virus titration and sequence analysis and to inoculate fresh Vero cells for the following passage. The *x*-axis shows the passage number and associated temperatures, and the *y*-axis shows the virus titer of the initial inoculum and passage harvests. Each symbol represents one lineage. *T*_SH_ indicates the shutoff temperature. The limit of detection is indicated by a dotted line.

The *ts/att* phenotype of the HMPV vaccine candidates relies on mutations analogous to the HRSV *“1030s*” and Δ1313/I1314L sites that had been extensively optimized for genetic and phenotypic stability. To evaluate the phenotypic and genetic stability of the corresponding versions of HMPV, we subjected C1238S, Y1246K, C1238S/Y1246K, and Δ1238 viruses to strong selective pressure by applying temperature stress during serial passage (Fig. 2). Vero monolayers in twelve replicate 25 cm^2^ flasks were infected with each HMPV mutant or wt HMPV at an MOI of 0.1 PFU/cell. As described in Materials and Methods, two replicate flasks were passaged serially at the permissive temperature of 32°C for 10 passages, serving as controls (Fig. 2A), and 10 replicate flasks for each virus were passaged serially at increasing temperature from 36°C to 40°C, with a 1°C increase every second passage, for a total of 10 passages and a total duration of up to about 70 days of culture (Fig. 2B through F). Each replicate flask represented a separate lineage. These conditions would favor the outgrowth of any viruses that lost temperature sensitivity.

At the permissive temperature of 32°C, all viruses replicated efficiently during 10 serial passages (Fig. 2A). Over 10 serial passages at temperatures increasing from 36°C to 40°C, all 10 lineages of wt HMPV and the C1238S mutant also replicated efficiently (Fig. 2B and C). Among the viruses designed to be temperature sensitive, the Y1246K virus (*T*_SH_ = 39°C) reached titers of at least 7.0 log_10_ PFU/mL through the second passage at 37°C, corresponding to physiological human body temperature. A gradual reduction in viral titers was observed at temperatures above 37°C; after passage 2 at 40°C, lineages 3, 4, 6, and 8 had no virus detectable; lineages 5, 7, 9, and 10 only reached low titers of approximately 3.6 log_10_ PFU/mL, indicating that replication was reduced by 2,500-fold compared to replicas grown at 32°C. Lineage 2 showed a 300-fold decrease in titer to 4.5 log_10_ PFU/mL (40°C, passage 2), and lineage 1 titers increased slightly at the second passage at 39°C before gradually decreasing to 5.4 log_10_ PFU/mL (40°C, passage 2), suggesting a small reduction of the *ts* phenotype in these two lineages.

All lineages of C1238S/Y1246K (*T*_SH_ = 38°C) and Δ1238 (*T*_SH_ = 39°C) replicated efficiently up to 37°C, with titers gradually decreasing at temperatures above 37°C, as expected based on their *T*_SH_. For C1238S/Y1246K (Fig. 2E), titers of six lineages (4–9) were below the limit of detection by the second passage at 40°C, while lineages 1, 2, 3, and 10 grew to low titers ranging from 3.3 (lineage 10) to 5.1 (lineage 3) log_10_ PFU/mL at the second passage at 40°C. Lineage 3 showed a slight rebound in titer from 4.7 to 5.1 log_10_ PFU/mL between passages 1 and 2 at 40°C. For the Δ1238 virus (Fig. 2F), viral titers of seven lineages (1, 3, 6, 7, 8, 9, and 10) were below the limit of detection at 40°C, while lineages 2, 4, and 5 still replicated to low titers at the second passage at 40°C. Thus, Y1246K, C1238S/Y1246K, and Δ1238 viruses remained completely or substantially restricted in replication under these stringent temperature stress conditions.

To evaluate the genetic stability after serial passage at permissive temperature, viral RNA was isolated from supernatants of both lineages of each virus (wt HMPV, C1238S, Y1246K, C1238S/Y1246K, and Δ1238) collected after the 10th passage at 32°C (up to about 70 days of culture), and the complete genomes were sequenced by Sanger sequencing from two overlapping RT-PCR products. The C1238S, Y1246K, and Δ1238 ts/att sites were stable in all lineages over 10 passages at 32°C. Overall, in progeny from 10 passages at 32°C, mutations in both lineages of a specific virus were identified at 13 sites (6 sites in wt HMPV; 2 sites in C1238S, 3 sites in Y1246K, and 1 site in C1238S/Y1246K and Δ1238), and mutations in one of two lineages were detected at 8 sites (Table 2). For example, in progeny from both lineages of the wt HMPV control, missense mutations in N and SH and silent mutations in the G and L ORFs were detected; in both lineages, subpopulations had acquired an additional adenosine residue in the G gene end signal, which would be considered phenotypically inconsequential. In C1238S progeny (both lineages), an A/C nt change in the M2 gene end signal was detected, and a nt insertion in the G ORF was detected in subpopulations, the latter leading to a frame shift at G codon 115 and introducing a stop codon at codon 139, shortening the G ORF by 61 codons. The attachment glycoprotein G is not essential for HMPV (25); indeed, the C1238S virus replicated effectively in Vero cells, irrespective of a subpopulation with this truncation. Progeny from both Y1246K lineages contained two missense mutations (K486R in the F ORF; Y93N in the SH ORF), and a TAG to AAG change, extending the M2-2 ORF (71 amino acids) by one codon (K72). Both C1238S/Y1246K lineages revealed a mutation in the N ORF (V218E); both lineages of Δ1238 had acquired a mutation in the L ORF, the only coding change observed in the L ORF in any virus after 10 passages at permissive temperature of 32°C (also present in Δ1238 progeny grown at higher temperatures). The presence of identical mutations in progeny from both lineages suggests that in the inoculum, small subpopulations may have been present below the detection level that became dominant during passage in Vero cells, even though no mutations were detected by Sanger sequencing in the original virus stocks.

**TABLE 2:** Nucleotide and amino acid substitutions of HMPV L mutants passaged at permissive temperature of 32°C for 10 passages in Vero cells

ORF or gene region^*a*^	nt mutation^*b*^	aa mutation^*c*^	Lineage (L) with the indicated mutation (*n* = 2)^*g*^				
			wt HMPV	C1238S	Y1246K	C1238S/Y1246K	Δ1238^*f*^
Leader region	A35T	–	–	–	L2	–	–
N	G68A	G5E	–	L2	–	–	–
N	A367G	N105D	–	–	L2	–	–
N	T707A	V218E	–	–	–	L1, 2	–
N	A1037G	N328S	L1, 2	–	–	–	–
M	A2512G	E111 (silent)	–	–	–	–	L2
M	T2826A	L216H	–	–	–	L1	–
F	A4523G	K486R	–	–	L1, 2	–	–
M2-2	G5446T	S71T	–	–	–	L1	–
M2-2	T5448A	Stop codon to 72K	–	–	L1, 2	–	–
M2 gene end	A5454C	–	–	L1, 2	–	–	–
SH gene start	A5473G	–	–	–	–	–	L1
SH	A5583G	K35E	L1, 2	–	–	–	–
SH	G5585T	K35N	–	–	–	L2	–
SH	T5757A	Y93N	–	–	L1, 2	–	–
G	6574.1 (+1A)^*d*^	T115 (frame shift)	–	L1, 2^*e*^	–	–	–
G	C6744T	I171 (silent)	L1, 2	–	–	–	–
G gene end	6929.1 (+1A)	–	L1, 2^*e*^	–	–	–	–
L	A8683G	V517 (silent)	L1, 2	–	–	–	–
L	T10993C	H1287 (silent)	L1, 2	–	–	–	–
L	C12401A	Q1757K	–	–	–	–	L1, 2

^*a*^ ORF, open reading frame. Gene regions: N, nucleoprotein; F, fusion protein; M2-2, M2-2 protein; SH, small hydrophobic protein; G, attachment glycoprotein; L, large polymerase. nt positions based on HMPV CAN97-83 (GenBank accession number AY297749).

^*b*^ To evaluate the genetic stability during serial passage at permissive temperature, viral RNA was isolated from supernatants of two lineages (lineage 1 [L1] and L2) of the indicated virus after 10 passages at 32°C, and the complete genomes (nt 21–13173, excluding the regions complementary to the outermost PCR primers) were sequenced by Sanger sequencing from two overlapping RT-PCR products. Nucleotide (nt) differences compared to the respective seed virus are indicated.

^*c*^ aa, amino acid position in the indicated open reading frame. N, nucleoprotein; F, fusion protein; M2-2, M2-2 protein; SH, small hydrophobic protein; G, attachment glycoprotein; L, large polymerase; L1 and L2, lineages 1 and 2. –, no amino acid change (non-coding region).

^*d*^ Single-nucleotide insertion at position 6574, resulting in a frameshift of the G ORF following codon 115 and truncation of the G ORF by 62 codons.

^*e*^ Mixed populations.

^*f*^ Lineage 2 of the Δ1238 virus additionally contained a cluster of T/C mutations (nt 5564, 5572, 5582, 5599, 5602, 5623, 5656, 5749, and 5957) in the SH gene, suggesting cytidine deaminase activity.

^*g*^ –, no mutation at this site.

The L ORF of progeny grown at higher temperatures was sequenced from the final passage in which the virus titer remained at or above 4 log_10_ PFU/mL, a threshold that allows for successful confirmation of viral genomic sequences (Fig. 2D through F; Table 3). For wt HMPV and the non-*ts* virus C1238S, five lineages were selected randomly and sequenced after the 10th passage (passage 2 at 40°C) (Fig. 2B and C). Of the five lineages of wt HMPV, three had a silent mutation at L codon F938 (also observed in one lineage passaged at 32°C), and one had a coding change (N491I) (Table 3). All five C1238S lineages retained the intended C1238S mutation, and only one coding change in the L ORF was detected in one out of five lineages (K1400M), indicating substantial genetic stability of the L ORF of these controls throughout the temperature stress test.

**TABLE 3:** Stability of the L ORF in HMPV *ts* mutants passaged at increasingly restrictive temperatures in Vero cells

nt mutation^*b*^	aa mutation^*c*^	Fraction of lineages with indicated mutation^*a*^				
		wt HMPV	C1238S	Y1246K	C1238S/Y1246K	Δ1238
A7600G	L156 (silent)	–	–	–	–	1/10 (L8)
T8086C	S318 (silent)	–	–	–	–	1/10 (L2)
A8366T	N412Y	–	–	–	1/10 (L1)	–
A8531G	N467D	–	–	–	–	1/10 (L4)
A8604T	N491I	1/5 (L8)		–	–	–
A8918C	K596Q	–	–	–	–	1/10 (L3)
A8964G	E611G	–	–	–	–	3/10 (L1, 2, 5)
C9490T	G786 (silent)	–	–	–	1/10 (L3)	–
A9698C	I856L	–	–	–	–	1/10 (L2)
T9946C	F938 (silent)	3/5 (L1, 5, 7)	–	–	–	–
G10604A	A1158T	–	–	8/10 (all except L1, 4)	1/10 (L3)	–
C10670A	Q1180K	–	–	–	10/10	1/10 (L1)
T10721G	L1197V	**–**	**–**	1/10 (L1)	–	–
A10790T	M1220L	–	–	–	1/10 (L10)	3/10 (L2, 5, 6)
**A10869T^*d*^**	**K1246I**	**–**	**–**	**–**	1/10 (**L2**)	**–**
T10993C	H1287 (silent)	5/5	–	–	–	–
A11331T	K1400M	–	2/5 (L4)	–	–	–
C12401A	Q1757K	–	–	–	–	10/10

^*a*^ To evaluate the genetic stability of the L ORF during serial passage at increasingly restrictive temperatures, viral RNA was isolated from progeny from the last passage in which the virus titer remained at or above 4 log_10_ PFU/mL (Fig. 2D through F). For HMPV wt control and C1238S, *n* = 5 of 10 lineages were randomly chosen for sequencing (wt HMPV; lineage 1 [L1], L5, L7, L8, and L10; C1238S; L2, L3, L4, L9, and L10); for the *ts/att* versions Y1246K, C1238S/Y1246K, and Δ1238, progeny from all 10 lineages were included. The genome region containing the L ORF was amplified by RT-PCR (nt 6991 through 13333) and sequenced by Sanger sequencing (nt 7011–13309, excluding regions complementary to primers). Lineage numbers with indicated mutations (L1–L10) are indicated in brackets. –, no mutation detected at this site.

^*b*^ Nucleotide (nt).

^*c*^ Amino acid (aa) positions of indicated mutations are based on HMPV CAN97-83 (Genbank accession number AY297749).

^*d*^ The adventitious point mutation A10869T at the *ts/att* Y1246K site in L1 of C1238S/ Y1246K and the resulting K1246I substitution are highlighted in bold.

For all lineages of the *ts* viruses Y1246K, C1238S/Y1246K, and Δ1238, progeny from the last passage in which the virus titer remained at or above 4 log_10_ PFU/mL stably retained the Y1246K, C1238S/Y1246K, and Δ1238 *ts/att* sites, except for a single lineage (L2) of C1238S/Y1246K that acquired a K1246I mutation (K1246I; AAA to ATA) (Table 3, bold). In a previous HRSV study, the amino acid assignment of isoleucine at the corresponding HRSV codon (Y1321I) was associated with a *T*_SH_ of 39°C, representing a partial (1°C) reduction in HRSV temperature sensitivity compared to the Y1321K assignment (*T*_SH_ = 38°C [42]). Thus, in the HMPV background of C1238S/Y1246K, the K1246I change in lineage 2 might have conferred a slightly higher level of resistance to temperature stress, but replication at higher temperatures remained highly restricted (Fig. 2E).

Amongst Y1246K progeny, lineage 1, which stood out in its residual ability to replicate at increased temperatures, had a nt substitution at position 10721, resulting in a conservative coding change (L1197V), unlikely to mediate a loss of temperature sensitivity. Except for lineages 1 and 4, all Y1246K lineages had a missense mutation A1158T in the L ORF, which did not seem to restore the ability of Y1246K to replicate at higher temperatures.

Among C1238S/Y1246K progeny, all 10 lineages had a point mutation at Q1180K, which, based on the apparent restriction of replication of the C1238S/Y1246K lineages above the *T*_SH_, did not reduce temperature sensitivity (Fig. 2E). Another missense mutation, A1158T, identified in 80% of Y1246K lineages, was also present in lineage 3 of C1238S/Y1246K, but based on the high level of restriction retained by Y1246K progeny with A1158T assignment, it is unlikely that this change was responsible for the residual ability of C1238S/Y1246K lineage 3 to replicate in Vero cells at higher temperatures.

As seen in the lineages grown at 32°C, for Δ1238 (*T*_SH_ = 39°C), all 10 lineages, including lineages with no virus detectable after the second passage at 40°C, had a non-silent point mutation at codon Q1757K. Additional missense mutations were detected in up to 30% of lineages of Y1246K, C1238S/Y1246K, or Δ1238, but none of these lineages regained the ability to replicate to wt-like titers at 40°C.

While the most prominent Q1180K (C1238S/Y1246K lineages) and Q1757K mutations (Δ1238 lineages) might have been induced by temperature stress, none of the lineages gained fitness to replicate at temperatures above their *T*_SH_ in Vero cells, suggesting that a compensatory effect of Q1180K or Q1757K was absent or minimal. Thus, extensive temperature stress over 10 sequential passages did not result in loss or reversion of the C1238S/Y1246K or Δ1238 sites, and silent and coding changes in the L ORF were minimal, indicating a high level of phenotypic and genetic stability, with no pattern of compensatory mutations.

### HMPV with *ts/att* mutations derived from HRSV vaccine candidates are restricted in replication but immunogenic and protective in hamsters

**Fig 3:**
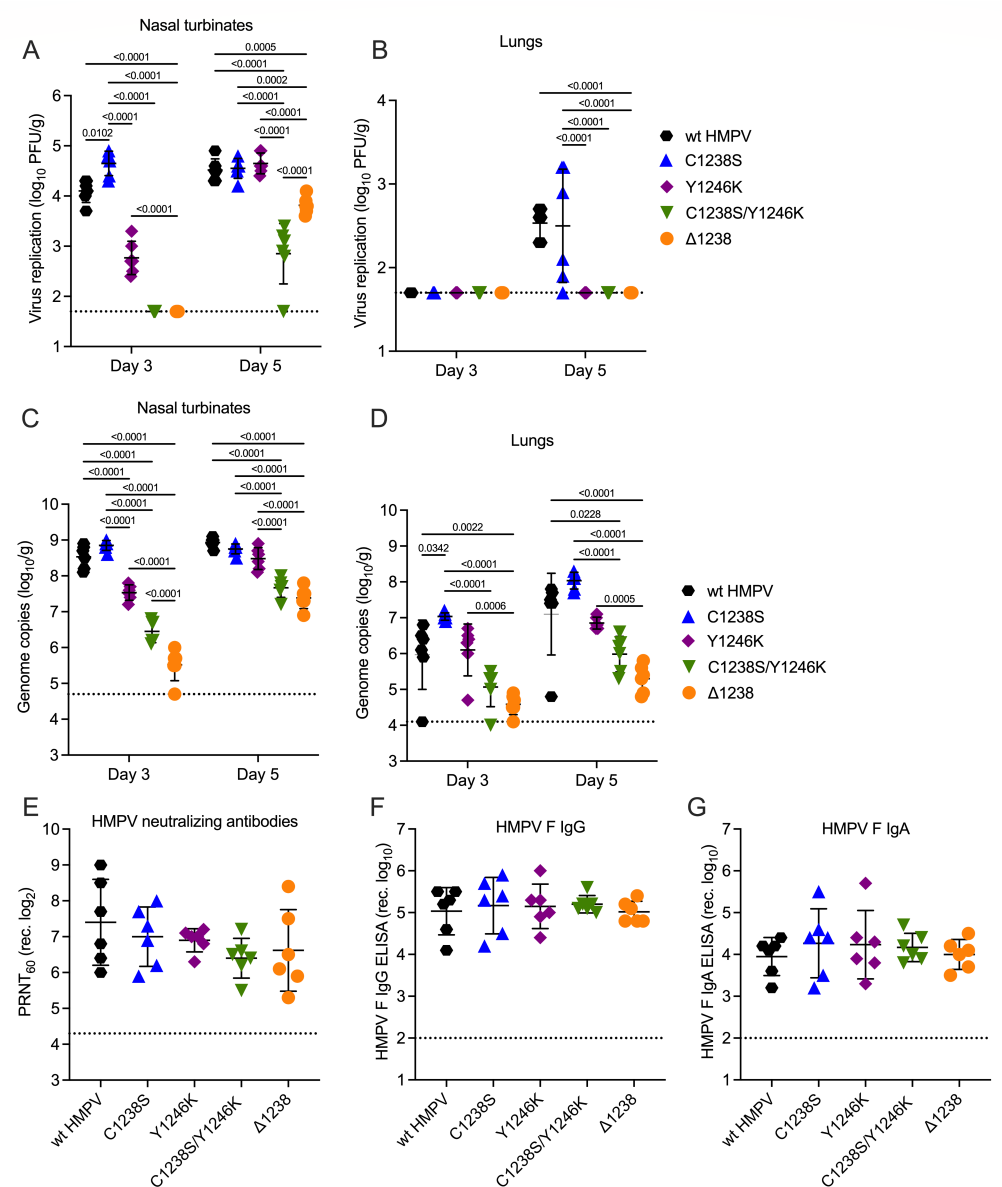
Replication and immunogenicity of HMPV L mutants in hamsters. Groups of 18 golden Syrian hamsters (5–6 weeks old) were inoculated intranasally with 10^5^ PFU of the indicated HMPV L mutants under isoflurane anesthesia. On days 3 and 5, six hamsters per group were euthanized, and nasal turbinates (NTs) and lungs were collected. The tissues were homogenized, clarified by centrifugation, and supernatants were snap frozen on dry ice. Virus titers of NTs (**A**) and lungs (**B**) were determined by plaque assay. The limit of detection (LOD, dotted line) was 50 PFU/g of tissue. For quantification of HMPV genomic RNA, viral RNA was extracted from tissue homogenates of NTs (**C**) and lungs (**D**) and quantified in triplicate by RT-qPCR using a HMPV P-specific TaqMan assay and expressed as log_10_ copies/g of tissue (LOD: 4.1 log_10_ copies/g and 4.7 log_10_ copies/g of lung and NT tissue, respectively). Standard curves were generated using serial dilutions of plasmids containing the targeted sequence. The means and SD are shown. Statistical differences are indicated (*n* = 6 per group per day, two-way ANOVA with Sidak post-hoc test). 60% HMPV plaque reduction neutralization titers (PRNT_60_) (**E**) and anti-HMPV F IgG (**F**) and IgA titers (**G**) of sera collected on day 28 post-immunization. Each symbol represents an individual hamster, with means and SD shown per group. No significant differences in the serology results were detected between the groups (*n* = 6 per group per day, two-way ANOVA with Sidak post-hoc test).

The replication, immunogenicity, and protective efficacy of the HMPV mutant viruses were also evaluated in hamsters. Golden Syrian hamsters were infected intranasally with 5.0 log_10_ PFU of wt HMPV or HMPV L derivatives. Virus replication in nasal turbinates (NTs) and lungs was evaluated on days 3 and 5 by plaque assay and by HMPV TaqMan assay to the HMPV P gene (six animals per group and time point, Fig. 3A through D). In NT, wt HMPV was detected at geometric mean titers (GMTs) of 4.1 log_10_ and 4.3 log_10_ PFU/g of tissue on days 3 and 5 (Fig. 3A). Among the viruses bearing mutations in the L protein, the non-ts virus C1238S replicated most efficiently, with a GMT of 4.5 log_10_ PFU/g in NT on day 3. By day 5 pi, GMTs in NT of wt HMPV and the non-ts C1238S virus, as well as the Y1246K virus, were comparable, while GMTs of Δ1238 and C1238S/Y1246K remained significantly lower (Fig. 3A). Upon quantification by TaqMan assay, similar geometric mean copy numbers (8–9 log_10_ copies/g for wt HMPV and C1238S) were detected in NT on both days. Copy numbers of Y1246K were significantly lower compared to those of the control viruses on day 3, and those of C1238S/Y1346K and Δ1238 were significantly lower on both days (Fig. 3C).

In lungs, HMPV titers were below the level of detection by immunoplaque assay in all groups on both days, except for wt HMPV and C1238S, which were detectable by titration on day 5 with GMTs of 2.4 log_10_ and 2.7 log_10_ PFU/g of tissue, respectively (Fig. 3B). TaqMan assay results revealed that in lungs, copy numbers of all viruses were lower than in NT; results for wt HMPV and C1238S were comparable, with no statistical difference on day 5 (Fig. 3C and D), while C1238S/Y1246K and Δ1238 were significantly more restricted in replication in lungs.

To determine the genetic stability of the attenuating sites during vaccine replication in hamsters, we isolated RNA from NT homogenates and performed RT-PCR to amplify a 1,584 bp region of the HMPV L gene (HMPV CAN97-83 nt 9805–11368) spanning the attenuating sites. Analysis by Sanger sequencing of nt 10470 through 11367 did not reveal any mutations in these regions, confirming the absence of reversions or changes at the attenuating sites following replication in hamsters. HMPV titers in lung tissues were too low to confirm genetic stability by sequence analysis.

From the remaining six hamsters per group, sera were collected at day 28 pi and the HMPV-neutralizing titers and anti-HMPV F IgG and IgA titers induced by these viruses were assessed (Fig. 3E through G). wt HMPV induced mean HMPV PRNT_60_ of 7.4 log_2_. The mean HMPV PRNT_60_ elicited by all viruses, including the two with the strongest *ts/att* phenotypes, C1238S/Y1246K and Δ1238, were comparable to those of wt HMPV and ranged between 6.4 (C1238S/Y1246K) and 7 log_2_ (C1238S). Anti-HMPV F IgG and IgA titers were also similar between all groups, with no differences between groups immunized with wt HMPV and HMPV with L mutations (Fig. 3E through G). Thus, despite the strong restriction in replication in hamsters of C1238S/Y1246K and Δ1238, these viruses were immunogenic in hamsters.

**Fig 4:**
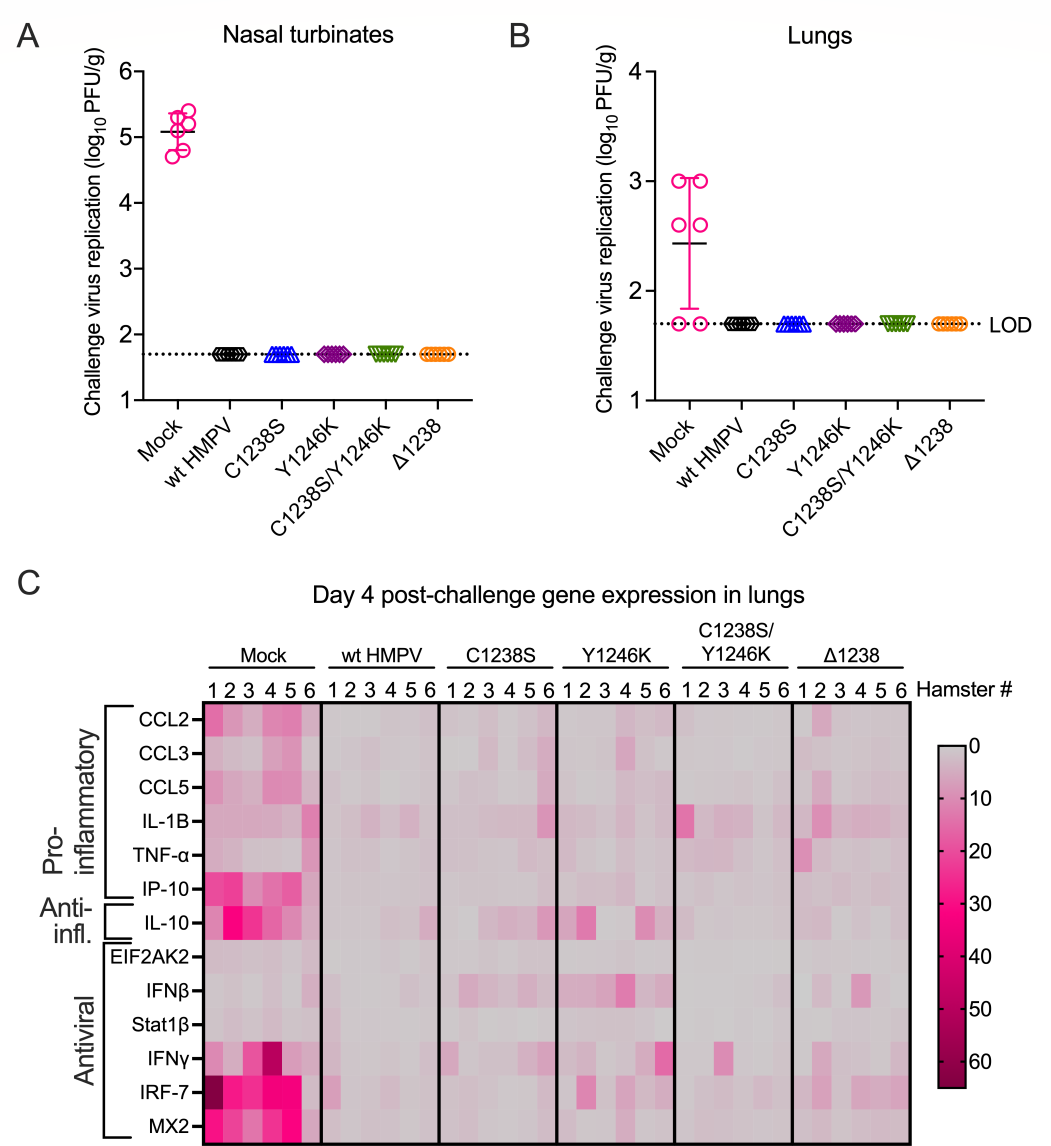
Protective efficacy of HMPV L mutants against wt HMPV challenge in hamsters. On day 30 pi, six hamsters from each immunized group were challenged intranasally with 6.5 log_10_ PFU of recombinant HMPV-SHs (wt HMPV). Six mock-immunized hamsters were challenged as controls. On day 4 post-challenge, all hamsters were euthanized, and NTs (**A**) and lungs (**B**) were collected, processed, and titered as described for Fig. 3 to evaluate challenge virus replication. Individual titers, means, and SD are shown for each group. Challenge virus replication was undetectable in NTs (*P* ≤ 0.0001 compared to mock-immunized animals) and lungs (*P* = 0.002 compared to mock-immunized animals) in all immunized groups (one-way ANOVA with Tukey post-test). (**C**) Total RNA was extracted from lung homogenates and subjected to TaqMan RT-qPCR to evaluate the expression of selected genes associated with inflammatory/antiviral responses. qPCR data were analyzed using the ΔΔCt method, normalized to β-actin, and expressed as fold-change over the average expression levels of five mock-immunized, mock-challenged hamsters of the same source and age group. The relative expression levels of antiviral and pro/anti-inflammatory genes on day 4 after challenge are shown as a heat map (*n* = 6 per group).

On day 30 pi, hamsters were challenged intranasally with 6.5 log_10_ PFU of wt HMPV. An additional group of mock-immunized animals was included to evaluate challenge virus replication. Four days after challenge, animals were sacrificed, nasal turbinates and lungs were harvested, and the level of wt HMPV challenge virus replication was assessed by immunoplaque assay. In the mock-infected control group, wt HMPV challenge virus titers of 5.2 log_10_ and 2.5 log_10_ PFU/g were detected in NT and lungs, respectively (Fig. 4A and B). Animals immunized with wt HMPV or HMPV bearing L mutations were protected, with no detectable wt HMPV challenge virus replication in the upper or lower respiratory tract.

To compare inflammatory responses among the immunized groups following wt HMPV challenge, the expression of 13 selected inflammatory/antiviral genes was analyzed by TaqMan real-time quantitative PCR (RT-qPCR) assays on total RNA extracted from lung homogenates 4 days post-challenge (pc) (Fig. 4C). qPCR data were normalized to β-actin and expressed as the fold-increase over the mean values from five unimmunized and unchallenged hamsters. IRF-7, MX2, IL-10, and IP-10 were the four most strongly upregulated antiviral/inflammatory genes in lungs from unimmunized control animals on day 4 post-HMPV challenge (31- and 21-, 16- and 15-fold increase on average on day 4 pc, respectively, Fig. 4C; Fig. S1). In contrast, these four genes were barely upregulated post-challenge in wt HMPV, C1238S, Y1246K, C1238/Y1246K, and Δ1238-immunized hamsters following wt HMPV challenge.

Thus, C1238S/Y1246K and Δ1238 are temperature sensitive, restricted in replication yet immunogenic and protective in hamsters, and the sites of the *ts/att* mutations are genetically stable *in vitro* and in hamsters. C1238S/Y1246K and Δ1238 represent promising live-attenuated vaccine candidates for intranasal immunization against HMPV.

## DISCUSSION

As a strategy to develop a live-attenuated intranasal HMPV vaccine for the pediatric population, we introduced two temperature-sensitive and attenuation (*ts/att*) mutations from HRSV into homologous positions of the HMPV L protein. The selected *ts/att* sites, “*1030s*” and Δ1313/I1314L, were originally identified in the L protein of leading HRSV vaccine candidates (42, 43, 46, 47). We chose this strategy because extensive research effort had been dedicated to the genetic stabilization of the *ts/att* mutations of the HRSV lead candidates, and the genetic stability of these sites was confirmed in several clinical studies. Genetic stability of *ts/att* mutations is essential for the safety of live-attenuated intranasal vaccine candidates. Given the high degree of amino acid and functional similarity in the specific domains between the L proteins of HRSV and HMPV, we hypothesized that these mutations would confer similar *ts/att* and stability properties to HMPV. Indeed, the resulting HMPV vaccine candidates displayed a *ts/att* phenotype, and the introduced mutations remained genetically stable during serial passage under temperature stress. In the golden Syrian hamster model, both candidates, C1238S/Y1246K and Δ1238, were attenuated *in vivo* while eliciting strong immune responses that conferred significant protection against challenge with wt HMPV. Thus, by transferring these specific *ts/att* codons from HRSV to HMPV, we were able to save substantial time in the preclinical development of HMPV vaccine candidates, and, most importantly, the high level of genetic stability of these sites originally developed in the HRSV background seemed to transfer to HMPV.

The polymerase proteins of HRSV and HMPV share approximately 45% amino acid identity. Both L proteins contain three conserved functional domains: the RNA-dependent RNA polymerase (RdRp) domain, the polyribonucleotidyl transferase (PRNTase or capping) domain, and the methyltransferase (MTase) domain, which catalyzes RNA cap methylation. In RSV vaccine candidates, the “*1030*” mutation (Y1321N) has been mapped to a region located between two critical motifs (motif C and the catalytic motif D) within the PRNTase domain (9). Structural analyses suggested that the tyrosine-to-asparagine substitution of HRSV L aa Y1321N (in case of the non-stabilized “*1030*” HRSV attenuating site [40]) or the substitution by a positively charged lysine residue (Y1321K) in the genetically stabilized HRSV version “*1030s*” may disrupt interactions between these motifs, altering the conformation of the catalytic motif. This structural change likely destabilizes the catalytic region, contributing to the temperature-sensitive phenotype observed in the HRSV “*1030*” virus, a phenotype that can be partially rescued at lower incubation temperatures (9). Based on the structural similarity, a similar attenuation mechanism may underlie the temperature sensitivity observed in the HMPV L mutant vaccine candidates carrying the corresponding Y1246K substitution. Since the Δ1238 deletion is in close proximity to the 1246 site, it might also destabilize this catalytic region of the L protein, with similar phenotypic consequences, resulting in a *ts* phenotype.

In this study, replication and temperature sensitivity of the HMPV vaccine candidates were evaluated using Vero cells supplemented with exogenous trypsin, required for cleavage of F protein to promote cell-to-cell fusion and virus spread (50). Vero cells are widely used for vaccine manufacture and are a commonly used cell line to amplify and study HMPV. The vaccine candidates replicated efficiently in Vero cells at the permissive temperature of 32°C. Upon sequencing after 10 serial passages at 32°C, the vaccine candidates remained genetically stable at the *ts/att* sites. In our study, 10 serial passages in Vero cells at permissive temperature resulted in a small number of point mutations in wild-type and mutant HMPV lineages. On the other hand, during the manufacture of pneumovirus vaccine candidates for clinical studies, cDNA-derived virus is recovered on Vero cells, and low-passage clinical study material is generated under well-defined conditions and using low MOIs, preventing issues related to potential genetic instability of these RNA viruses during extended passage.

During serial passage at increasingly higher temperature in an extended *in vitro* stress test, the ability of the wt HMPV and the non-*ts* C1238S controls to replicate was barely affected by temperatures increasing from 32°C up to 40°C. For temperature-sensitive versions Y1246K, C1238S/Y1246K, and Δ1238, the total duration of replication during serial passages was up to 70 days. For C1238S/Y1246K and Δ1238, 60% and 70% of the lineages had no virus detectable after the second passage at 40°C. Sequence analysis of the L ORFs of all 10 lineages revealed some missense and silent mutations that had accumulated under temperature stress at a number of sites. However, except for a single lineage with a Y1248I substitution, which, in HRSV, had only a minor de-attenuating effect, none of the mutations were located at sites of *ts/att* mutations, suggesting that the transfer of the HRSV *ts/att* substitutions to HMPV resulted in genetically stable attenuating sites. Polymerases of RNA viruses lack proofreading ability and sustain mutations at a frequency of approximately 10^−4^, which is approximately equivalent to one mutation per HMPV genome, allowing for rapid outgrowth of variants in response to selective pressure such as temperature stress. The accumulation of mutations during passage of HMPV in Vero cells has been previously observed, in particular in the SH and G genes and the M2 gene end signal, even at the permissive temperature of 32°C (49). Upon *in vitro* stress at increasing temperatures, both *ts/att* vaccine candidates, C1238S/Y1246K and Δ1238, each acquired an additional missense mutation in all 10 lineages in the polymerase L protein, resulting in substitutions Q1180K and Q1757K, respectively. In each case, a neutrally charged glutamine (Q) was replaced by a positively charged lysine (K). However, these charge-shifting mutations from Q to K alone do not appear to impact temperature sensitivity, as neither of the mutants acquired the ability to produce progeny virus when grown at 40°C for two passages in the absence of other adventitious mutations in the L ORF.

Even though hamsters are not fully permissive for HMPV, they are susceptible to HMPV infection and replication in the respiratory tract. The immunogenicity of HMPV in hamsters can serve as a correlate for immunogenicity in humans (28, 51), and restriction of replication of HMPV vaccine candidates in the upper and lower respiratory tract of hamsters serves as a correlate for attenuation in fully permissive hosts. Upon intranasal inoculation, C1238S/Y1246K and Δ1238 were highly restricted in the upper and lower airways. The restriction in replication is expected to correlate with attenuation in humans.

Despite the restricted replication in the lungs of C1238S/Y1246K and Δ1238, a single intranasal dose was highly immunogenic in hamsters. Robust HMPV-neutralizing serum antibody titers were observed in all immunized hamsters. In addition, C1238S/Y1246K and Δ1238 elicited similar serum IgG and IgA titers to the HMPV F protein as wt HMPV; despite their restricted replication, C1238S/Y1246K and Δ1238 were immunogenic and protective in hamsters against wt HMPV challenge. The low or undetectable inflammatory response in the lungs of C1238S/Y1246K and Δ1238 immunized hamsters following challenge with wt HMPV further confirmed efficient protection. Based on these results, HMPV C1238S/Y1246K and Δ1238 represent promising live-attenuated HMPV vaccine candidates, predicted to be attenuated and immunogenic. The temperature-sensitivity phenotype conferred by the *ts/att* sites provides a safeguard against vaccine reactogenicity in the lower respiratory tract, and the underlying mutations are expected to be genetically stable.

## MATERIALS AND METHODS

### Cell lines

African green monkey kidney (Vero) cells were maintained at 37°C with 5% CO_2_ in Opti-MEM (Gibco-Life Technologies) supplemented with 5% fetal bovine serum (FBS, HyClone) and 1% l-glutamine (Gibco-Life Technologies). Baby hamster kidney (BSR T7/5) cells constitutively expressing the T7 RNA polymerase (52) were grown in GMEM media (Gibco-Life Technologies) supplemented with 10% FBS and 2% MEM amino acids (Gibco-Life Technologies). For every other passage, the medium was supplemented with 2% of geneticin (Quality Biological) to maintain the stability of expression of T7 polymerase.

### Construction and generation of recombinant HMPV mutants from cDNA

Recombinant HMPV was generated using a reverse genetics system for strain CAN97-83 (GenBank accession number AY297749). A full-length cDNA plasmid containing the rHMPV-SHs antigenome, with the HMPV SH ORF stabilized to prevent frequent frame-shift mutations observed in Vero cells (49), was used to generate the HMPV L mutants. Mutations were introduced into the antigenome cDNA by site-directed mutagenesis (QuikChange Lightning Site-Directed Mutagenesis Kit, Agilent Technologies) per the manufacturer’s recommendation. Recombinant HMPV with these changes was recovered by reverse genetics as previously described (50). Briefly, confluent BSR T7/5 cells were transfected using Lipofectamine 2000 (Thermo Fisher Scientific) with HMPV full-length cDNA plasmids with the desired mutations and four support plasmids, pTM(N), pTM(P), pTM(M2-1), and pTM(L), which supply the HMPV nucleocapsid protein (N), phosphoprotein (P), M2-1 protein, and large polymerase protein (L). Transfected cells were incubated at 32°C for at least 24 h. The BSR T7/5 cells were harvested by scraping into the medium, and the cells and medium were then added to sub-confluent monolayers of Vero cells in Opti-MEM medium containing 1% l-glutamine and incubated at 32°C. HMPV propagation in cell culture relies on added trypsin to mediate activation cleavage of the HMPV F protein. Trypsin (TrypLE Select, Gibco) was added to Vero cells at a final concentration of 4% and replenished every second day. Viruses were harvested 9–13 days post-transfection by scraping the cells into the supernatant. Cells and supernatants were vortexed to detach cell-associated virus particles. Supernatants were clarified by centrifugation and snap-frozen. Virus titers were determined by immunoplaque assay, and viruses were passaged one more time at low multiplicity of infection (MOI) on Vero cells in the absence of serum and in the presence of 4% of TrypLE Select (replenished every other day) to generate passage 2 (P2) working stocks. Viral RNA was isolated, and the complete sequence of each viral genome was determined by Sanger sequencing of overlapping PCR products. The only sequences that were not directly confirmed for each genome were the regions complementary to the outermost primers, namely, nt 1–20 and 13174–13335.

### HMPV titration by immunoplaque assay

Virus titers were determined by immunoplaque assay on Vero cells. Sub-confluent Vero cells in 24-well plates were washed twice with Opti-MEM to remove FBS and inoculated in duplicate with 10-fold serial dilutions of HMPV test samples. The plates were rocked for 2 h at 32°C, the inoculum was removed, and monolayers were overlaid with Opti-MEM containing 0.8% methylcellulose, 1% l-glutamine (Gibco-Life Technologies), 2.5% penicillin-streptomycin, 0.1% gentamycin, 0.5% amphotericin B, and 4% TrypLE Select. On day 3, a second overlay was added to replenish TryPLE Select. After 7–10 days, the cells were fixed overnight with ice-cold 80% methanol, and plaques were visualized by immunostaining with a mixture of two HMPV F-specific human monoclonal antibodies (MPE8 and MPF5 [53, 54]), followed by incubation with infrared dye (IRD)-conjugated goat anti-human IgG (LICOR). After extensive washing, plates were scanned with an Odyssey CLx imager (LI-COR).

### Multicycle growth kinetics of HMPV L mutants

Sub-confluent monolayers of Vero cells in six-well plates were washed twice with Opti-MEM to remove FBS and infected with the indicated viruses at an MOI of 0.01 PFU/cell. After a 3-h virus adsorption, the inoculum was replaced with fresh medium containing 1% l-glutamine and 4% TrypLE Select. One set of plates was incubated at 32°C, while the other set was incubated at 37°C. Duplicate wells of each virus grown at each temperature were harvested at 24 h intervals by scraping the cells into the medium. The cell and medium suspensions were vortexed briefly three times, and supernatants were clarified by centrifugation at 300 × *g* for 5 min at 4°C. Aliquots were snap-frozen in dry ice and stored at −80°C before titration by immunoplaque assay as described above.

### Evaluation of the *ts* phenotype of HMPV vaccine candidates

The *ts* phenotype of each of the HMPV mutants was determined by evaluation of plaque formation at 32°C, 35°C, 36°C, 37°C, 38°C, 39°C, and 40°C. Plaque assays were done on Vero cells as described above, and replica 24-well plates were incubated in sealed caskets at various temperatures in temperature-controlled water baths as previously described (55). The plates were incubated for 7 days, methanol fixed, and plaques were visualized by immunostaining (see immunoplaque assay).

### Genetic stability of HMPV L mutants in cell culture

For serial passages, 12 replicates of Vero cells in 25 cm^2^ flasks were infected with wt HMPV or indicated HMPV mutants at an MOI of 0.1 PFU/cell. Each replicate represented an independent lineage. Prior to infection, Vero cells were washed twice to remove FBS. After a 3-h adsorption, the inoculum was replaced with Opti-MEM containing 1% l-glutamine and 4% TrypLE Select. Two lineages were maintained at 32°C for 10 serial passages (controls), while the remaining 10 lineages were serially passaged at progressively higher temperatures, increasing by 1°C every second passage from 36°C to 40°C, for a total of 10 passages. Infected cultures were harvested when the cytopathic effect was extensive (4–7 days post-infection). Cells were scraped into the medium and vortexed; supernatants were clarified by low-speed centrifugation, aliquoted, and snap-frozen on dry ice for titration and sequence analysis. For each subsequent passage, 0.5 mL of clarified supernatant was used to inoculate a fresh 25 cm^2^ flask of Vero cells.

The genomic sequences of progeny from serial passages were determined by Sanger sequencing of overlapping PCR fragments. Briefly, viral RNA was extracted using the QIAamp Viral RNA extraction kit (Qiagen) from clarified supernatants collected at the end of passage 10 for control lineages grown at 32°C. For the stressed lineages, viral RNA was extracted from supernatants collected after the last passage, reaching a titer at or above 4 log_10_ PFU/mL. HMPV cDNA was generated by reverse transcription using the Maxima H Minus First Strand cDNA Synthesis Kit (Thermo Fisher). The cDNA was then amplified in overlapping PCR fragments using the SequalPrep Long PCR Kit with HMPV-specific primers and added dNTPs (Thermo Fisher). PCR products were purified and sequenced by Sanger sequencing.

### Replication, immunogenicity, and protective efficacy in hamsters

Animal studies were approved by the NIAID Animal Care and Use Committee (ACUC). Male golden Syrian hamsters (*Mesocricetus auratus;* Envigo Laboratories, Frederick, MD), 5–6 weeks of age, were randomly divided into groups of 18 animals. Serum was collected before inoculation. Hamsters were immunized intranasally on day 0 with 5.0 log_10_ PFU of the indicated viruses, diluted in Opti-MEM (100 µL volumes per animal; 50 µL per nostril) under light isoflurane anesthesia. On days 3 and 5 post-immunization, six hamsters per group were euthanized by CO_2_ inhalation, and lungs and nasal turbinates were harvested. Tissues were weighed, and 10 mL/g of tissue of L-15 medium was added. Tissues were homogenized using a gentleMACS Dissociator (Miltenyi Biotech), and homogenates were clarified by centrifugation. Supernatants were aliquoted, snap-frozen in dry ice, and stored at −80°C. Virus titers were evaluated later by immunoplaque assay, as described above. From the remaining hamsters, serum was collected on day 28 post-infection to evaluate the HMPV-specific antibody response (*n* = 6). Hamsters were challenged on day 30 pi intranasally with 6.5 log_10_ of recombinant wt HMPV (rHMPV-SHs [49]) in 100 µL volumes. An additional group of mock-immunized hamsters (*n* = 6) was included in the HMPV challenge. On day 4 post-challenge, hamsters were euthanized, and the nasal turbinates and lungs were collected and processed as described above.

To determine the HMPV neutralizing antibody titers, sera were evaluated by 60% plaque-reduction neutralization assay in the absence of complement. Sera were heat-inactivated at 56°C for 30 min. Serially diluted sera were mixed with equal volumes of medium containing rHMPV-SHs and incubated at 37°C for 1 h. The mixtures were added to Vero cell monolayers in duplicates, incubated for 2 h, washed, and overlaid with Opti-MEM containing methylcellulose as described above (immunoplaque assay). After 6–7 days of incubation at 37°C, plates were fixed and immunostained as described (immunoplaque assay). The 60% plaque reduction neutralizing antibody titers (PRNT_60_) were calculated.

### Quantification of HMPV RNA expression and expression of inflammatory/antiviral genes in hamster tissues by qRT-PCR

Homogenized tissues were mixed with TRIzol LS Reagent (Thermo Fisher), and RNA was extracted using the Phasemaker Tubes Complete System (Thermo Fisher) and the PureLink RNA Mini Kit (Thermo Fisher) following the manufacturer’s instructions.

Absolute quantification of HMPV RNA was performed using the TaqMan RNA-to-Ct 1-Step Kit (Thermo Fisher) using a TaqMan assay specific to the HMPV P gene of strain HMPV CAN97-83 described previously (56). The assay was performed on a QuantStudio 7 Pro real-time PCR system (Thermo Fisher). Copy numbers were determined using standard curves generated using serially diluted plasmids encoding the target sequence. The sensitivity of the TaqMan assay was 10 copies per reaction, corresponding to a limit of detection of 4.1 log_10_ copies and 4.7 log_10_ copies/g of lung and nasal turbinates tissue, respectively.

Relative quantification of the expression of inflammatory/antiviral genes in lung tissues was performed using custom-ordered TaqMan low-density array cards (Thermo Fisher Scientific). Cards contained TaqMan primers and probes for inflammatory/antiviral genes of golden Syrian hamsters, and β-actin was included as a housekeeping gene (57–60). cDNA was synthesized using the High-Capacity RNA-to-cDNA Kit (Thermo Fisher Scientific) from 7 µL of RNA extracted from lung homogenates. cDNA was mixed with TaqMan Fast Advanced Master Mix (Thermo Fisher), and real-time PCR was performed in triplicate wells for each target and each sample on the QuantStudio 7 Pro (Thermo Fisher). Results were analyzed using the comparative threshold cycle (ΔΔCT) method, normalized to β-actin, and expressed as fold changes over the average expression in five non-immunized, non-challenged hamsters.

### Expression and purification of the HMPV F protein

The HMPV F protein was expressed using a pVRC8400 plasmid. The plasmid encodes amino acids 1–524 of the HMPV F protein of strain CAN97-83, followed by a fibritin trimerization domain (foldon) from the T4 phage, a thrombin cleavage site, a 6× His tag, and a Strep-II tag. The plasmid was transfected into Expi293 cells using ExpiFectamine. On day 6 post-transfection, the supernatant containing the F protein was harvested and clarified by centrifugation, followed by filtration through a 0.2 μm PES membrane. The clarified supernatant was then mixed with Ni-NTA Agarose resin (Thermo Fisher) for 3 h at 4°C with slow rotation. The mixture was poured onto a gravity flow column (Bio-Rad) and washed five times with 1× DPBS (Gibco). The F protein was then eluted using 1× DPBS containing 0.5 M imidazole, further concentrated using an Amicon filter, and purified by size-exclusion chromatography in 1× DPBS using a Superdex 200 Increase 10/300 GL column (Cytiva). Aliquots of the purified HMPV F protein were snap-frozen in liquid nitrogen and stored at −80°C until use.

### Dual IgG- and IgA-specific dissociation-enhanced lanthanide fluorescence immunoassay (DELFIA) ELISA

IgG and IgA antibodies to the HMPV F protein were determined by IgG- and IgA-specific dissociation-enhanced lanthanide fluorescent (DELFIA) time-resolved fluorescence (TRF) immunoassay, similar to the HRSV F and G ELISAs described previously (61). In brief, black 96-well plates (Thermo Fisher) were coated with HMPV F antigen (100 ng/well) overnight at 4°C, washed once with 1× DPBS (Gibco) containing 0.1% IGEPAL CA-630 (Millipore Sigma), and blocked with 5% (wt/vol) dry milk powder overnight at 4°C. Serial threefold dilutions of serum samples were transferred to the antigen-coated plates in duplicate and incubated for 1 h, followed by incubation with a cocktail of secondary antibodies [goat anti-hamster IgG(H+L)-HRP (Invitrogen)] and rabbit anti-hamster IgA-biotin (Brookwood Biomedical). After 1-h incubation at room temperature, plates were washed, diluted streptavidin-europium (PerkinElmer) was added, incubated for 1 h, and plates were washed three times as above. To detect IgG, Pierce ECL substrate (Thermo Fisher) was added to all wells, incubated for 10 min, and plates were read on a Synergy neo plate reader (BioTek) for luminescence. After the read, plates were washed again, pre-warmed enhancement solution (PerkinElmer) was added, and plates were further incubated for 20 min. To detect IgA signals in the same wells, plates were read again using settings for time-resolved streptavidin-europium fluorescence (excitation wavelengths of 360/40 nm; emission filters of 620/40 nm). For data processing, the mean reading from duplicate blank sample wells from each read was subtracted from the mean reading from duplicate wells for each sample of that read. The cut-off was set to the blank mean plus three standard deviations of blank readings. The IgG and IgA titers of each sample were then determined by interpolating the sigmoid standard curves generated on GraphPad Prism version 9.5.

### Statistical analysis

Data sets were analyzed for significance using one-way ANOVA with Tukey post-test or two-way ANOVA with Sidak’s post-test on GraphPad Prism version 9.5. A log_10_ transformation was applied to data sets when necessary to obtain comparable standard deviation among groups of values. Data sets were only considered significantly different at *P* ≤ 0.05.

## Data Availability

All data supporting the findings of this study are available within the article and its supplemental material.

## SUPPLEMENTAL MATERIAL

Figure S1 jvi.00692-26-s0001.docx Gene expression in lung tissue (corresponding to Fig. 4).

ASM does not own the copyrights to Supplemental Material that may be linked to, or accessed through, an article. The authors have granted ASM a non-exclusive, world-wide license to publish the Supplemental Material files. Please contact the corresponding author directly for reuse.
