# Supplementary material for: Live human metapneumovirus vaccine candidates attenuated by temperature sensitivity mutations from human respiratory syncytial virus
Source: J Virol. 2026 Jul 6;100(8):e00692-26. doi: 10.1128/jvi.00692-26 (PMC13483368; doi:10.1128/jvi.00692-26)
Supplement: Supplementary file 1 [file jvi.00692-26-s0001.docx]

**Supplemental Material**

**Figure S1: Gene expression in lung tissue (corresponding to Figure 4).** On day 30 p.i., six hamsters from each immunized group were challenged with 6.5 log_10_ PFU of recombinant HMPV-SHs (wt HMPV). Six mock-immunized hamsters were challenged as controls. On day 4 post-challenge, all hamsters were euthanized; lungs were collected and processed as described for Figure 4C. Total RNA was extracted from lung homogenates and subjected to TaqMan RT-qPCR to evaluate the expression of selected genes associated with inflammatory/antiviral responses. qPCR data were analyzed using the ΔΔCt method, normalized to β-actin, and expressed as fold-change over the average expression levels of five mock-immunized, mock-challenged hamsters. The relative expression levels of interferon regulatory factor 7 (IRF7), MX2, IP10, and interleukin 10 (IL10) genes on day 4 after challenge are indicated (n = 6 per group). Each symbol represents an individual hamster and means and SD for each group are represented by lines.
